# Perivascular spaces as a potential biomarker of Alzheimer’s disease

**DOI:** 10.3389/fnins.2022.1021131

**Published:** 2022-10-18

**Authors:** Miranda Lynch, William Pham, Benjamin Sinclair, Terence J. O’Brien, Meng Law, Lucy Vivash

**Affiliations:** ^1^Department of Neuroscience, Central Clinical School, Monash University, Melbourne, VIC, Australia; ^2^Department of Neurology, Alfred Hospital, Melbourne, VIC, Australia; ^3^Department of Medicine, Royal Melbourne Hospital, University of Melbourne, Melbourne, VIC, Australia; ^4^Department of Neurology, Royal Melbourne Hospital, University of Melbourne, Melbourne, VIC, Australia; ^5^Department of Radiology, Alfred Health, Melbourne, VIC, Australia; ^6^Department of Electrical and Computer Systems Engineering, Monash University, Melbourne, VIC, Australia

**Keywords:** perivascular space (PVS), glymphatic system, Alzheimer’s disease (AD), amyloid-beta, tau, APOE4, Aquaporin 4 (AQP4), enlarged perivascular spaces (ePVS)

## Abstract

Alzheimer’s disease (AD) is a highly damaging disease that affects one’s cognition and memory and presents an increasing societal and economic burden globally. Considerable research has gone into understanding AD; however, there is still a lack of effective biomarkers that aid in early diagnosis and intervention. The recent discovery of the glymphatic system and associated Perivascular Spaces (PVS) has led to the theory that enlarged PVS (ePVS) may be an indicator of AD progression and act as an early diagnostic marker. Visible on Magnetic Resonance Imaging (MRI), PVS appear to enlarge when known biomarkers of AD, amyloid-β and tau, accumulate. The central goal of ePVS and AD research is to determine when ePVS occurs in AD progression and if ePVS are causal or epiphenomena. Furthermore, if ePVS are indeed causative, interventions promoting glymphatic clearance are an attractive target for research. However, it is necessary first to ascertain where on the pathological progression of AD ePVS occurs. This review aims to examine the knowledge gap that exists in understanding the contribution of ePVS to AD. It is essential to understand whether ePVS in the brain correlate with increased regional tau distribution and global or regional Amyloid-β distribution and to determine if these spaces increase proportionally over time as individuals experience neurodegeneration. This review demonstrates that ePVS are associated with reduced glymphatic clearance and that this reduced clearance is associated with an increase in amyloid-β. However, it is not yet understood if ePVS are the outcome or driver of protein accumulation. Further, it is not yet clear if ePVS volume and number change longitudinally. Ultimately, it is vital to determine early diagnostic criteria and early interventions for AD to ease the burden it presents to the world; ePVS may be able to fulfill this role and therefore merit further research.

## Introduction

Alzheimer’s disease (AD) is the most common form of dementia, with approximately 10 million people diagnosed each year globally (Iqbal et al., 2010; World Health Organization, 2017). It is anticipated that there will be approximately 150 million cases of dementia worldwide by 2050, with 60–80% likely attributed to AD (Nichols et al., 2022). Despite this, no effective treatments are available, and AD diagnosis is confirmed only on autopsy (Wren et al., 2018). AD presents an enormous societal and economic burden globally, and the critical question is how early pathology can be identified and how potential treatments can promote positive patient outcomes.

Alzheimer’s disease proteins have long been the target of AD research, most notably Amyloid-β (Aβ) and tau. Recent research has determined that while these proteins are implicated in the pathophysiology of AD, mechanisms by which Aβ and tau accumulate in the brain are thought to be due to the impaired clearance of proteins and other toxins by the glymphatic system (Jessen et al., 2015), and its Perivascular Spaces (PVS). Further investigation is needed regarding this system’s role in AD pathophysiology as a diagnostic marker and a potential therapeutic target in AD (and other proteinopathies).

## Alzheimer’s disease

Diagnosis of a cognitive disorder requires the patient to present with progressive impairment in one or more cognitive domains (Petersen et al., 1999). Mild cognitive impairment (MCI) is the transitional stage between normal cognition and impaired cognition (dementia) (Petersen et al., 1999). According to the diagnostic criteria, individuals are diagnosed with MCI when presenting with cognitive changes; however, functional abilities remain (Hugo and Ganguli, 2014). When impairment progresses to a level with a significant impact on social or occupational function, a dementia diagnosis is given (McKhann et al., 2011). AD diagnosis occurs when the patient presents with severe progressive impairment in several cognitive domains, which interfere with everyday functioning and independence, as well as positive to the known AD biomarkers (McKhann et al., 2011). The certainty of an AD diagnosis can be increased by supporting biomarker data—the ATN classification, as shown in Table 1. This system was designed to contain two biomarkers in each category, a Cerebrospinal Fluid (CSF) and an imaging biomarker (Jack et al., 2018). These biomarkers have been included to allow the use of the ATN classification system when there was imaging or biomarker data available, without needing both (Jack et al., 2018). Patients can be diagnosed with possible or probable AD depending on the symptom presentation (Table 2). Furthermore, as described in Table 3, Biomarkers can further support a diagnosis of AD (or other dementia) (Jack et al., 2018).

**TABLE 1:** ATN biomarker classification was established by Jack et al. (2018).

A	Aggregated Aβ	Reduced CSF Aβ42 or CSF Aβ42/Aβ40 ratio Increased binding on Aβ-PET
T	Aggregated Tau (NFTs)	Increased CSF phosphorylated tau Increased binding on Tau-PET
N	Neurodegeneration and neuronal injury	Brain atrophy on MRI Reduced brain metabolism FDG-PET Increased CSF total tau

The classification includes an imaging and CSF option to allow maximum inclusion if patient data is missing (Jack et al., 2018).

**TABLE 2:** Criteria for an Alzheimer’s disease (AD) diagnosis including biomarkers.

**Diagnostic category**	**Biomarker probability of AD etiology**	**Aβ (PET or CSF)**	**Neuronal injury (CSF tau, FDG-PET, structural MRI)**
**Probable AD dementia**			
- Based on clinical criteria	Uninformative	Unavailable, conflicting, or indeterminate	Unavailable, conflicting, or indeterminate
- With three levels of evidence of AD pathophysiological process	Intermediate Intermediate High	Unavailable or indeterminate Positive Positive	Positive Unavailable or indeterminate Positive
**Possible AD dementia (atypical clinical presentation)**			
- Based on clinical criteria	Uninformative	Unavailable, conflicting, or indeterminate	Unavailable, conflicting, or indeterminate
- With evidence of AD pathophysiological process	High but does not rule out second etiology	Positive	Positive
**Dementia-unlikely due to AD**	Lowest	Negative	Negative

Diagnosis of AD falls into Probable, Possible, or Unlikely. Table adapted from McKhann et al. (2011).

**TABLE 3:** Alzheimer’s disease (AD) profiles and categories depending on biomarker status.

AT(N) profiles	Biomarker category		
A-T-(N)-	Normal AD biomarkers		
A+T-(N)-	Alzheimer’s pathologic change	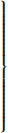	
A+T+(N)-	Alzheimer’s disease		
A+T+(N)+	Alzheimer’s disease		Alzheimer’s Disease Continuum
A+T-(N)+	Alzheimer’s and concomitant suspected non − Alzheimer’s Pathologic change			
A-T+(N)-	Non-AD pathologic change		
A-T-(N)+	Non-AD pathologic change		
A-T+(N)+	Non-AD pathologic change		

Bracket indicates AD diagnosis that lies on the Alzheimer’s disease Continuum. Alzheimer’s continuum is a diagnosis of AD or AD pathologic change. Adapted from Jack et al. (2018).

Pathologically, AD is characterized by Aβ plaques, neurofibrillary tangles (NFTs), and atrophy (Kinney et al., 2018). It is currently understood that Aβ plaques develop over many years without associated or significant cognitive decline (Bouras et al., 1994; Braak and Braak, 1997; Caselli and Reiman, 2013), some suggesting Aβ plaque build-up occurs up to 20 years before dementia onset (McDade et al., 2018), before seeming to reach a plateau as cognitive symptoms present (Sperling et al., 2011). In comparison, the accumulation of tau, phosphorylated-tau (p-tau), and NFTs tend to coincide with the presentation of cognitive issues (Hock et al., 1995; Buchhave et al., 2012).

Brain atrophy, beyond what is associated with normal aging, is also seen in AD (Miller et al., 1980; Silbert et al., 2003). Ventricular enlargement and volume loss occur in temporal gray matter and orbitofrontal and temporal cortices, including the hippocampus, and act as reliable biomarkers of AD progression (Silbert et al., 2003; Driscoll et al., 2009; Schuff et al., 2009). Hippocampal volume loss appears most characteristically in MCI individuals and can predict the likelihood of AD progression (Henneman et al., 2009). Notably, Desikan et al. (2011) demonstrated that global Aβ-associated volume loss only occurred in the presence of p-tau (Desikan et al., 2011). Subsequent studies showed that accelerated decline from MCI into dementia only occurred with the presence of p-tau (Desikan et al., 2011). Such findings suggest that there is a synergistic interaction between Aβ and p-tau that promotes volume loss and therefore accelerates cognitive decline more significant than what is seen in normal aging individuals.

### Amyloid-beta pathology

Amyloid-β, a primary pathological hallmark of AD, exists as a transmembrane protein in the brain and is produced from the cleaving of the amyloid precursor protein (APP) (Fan et al., 2020). APP possesses a single membrane spanning domain, a large N-terminus and short C-terminus (Chen et al., 2017). APP undergoes two proteolytic cleavages, resulting in different fragments with differing pathologies (Vaillant-Beuchot et al., 2020). APP cleaved first by β-secretase produces N- and C-terminal moieties 43, 45, 46, 48, 49, and 51 amino acids in length (Checler, 1995; Olsson et al., 2014). These moieties are again cleaved by γ-secretase in the endocytic compartment to form Aβ40 and Aβ42 (Olsson et al., 2014). These final Aβ monomers can aggregate in a number of ways, including oligomers, protofibrils and Aβ fibrils (Chen et al., 2017). Aβ fibrils are insoluble and large and can further aggregate in Aβ plaques, while Aβ oligomers are soluble and spread globally through the brain (Chen et al., 2017). Several studies have suggested that truncated forms of Aβ aggregate and correlate with disease severity and progression (Piccini et al., 2005; Güntert et al., 2006).

It has been suggested that microglia play a key role in the degradation of both Aβ forms (Soto-Rojas et al., 2021). And it is further postulated that this degradation may cause the activation or production of toxic molecules that could then go on to affect the Blood-Brain Barrier (BBB) or PVS, promoting disease progression (Moore et al., 2002; Merlo et al., 2020; Soto-Rojas et al., 2021).

Genetic forms of AD, which lead to early onset, are associated with mutations in APP or Presenilin 1/2, exacerbating Aβ accumulation (Ryan and Rossor, 2010). This knowledge suggests that Aβ mutations or dysfunction promote AD onset; therefore, therapies targeting Aβ removal would prevent disease onset. As such, the removal of Aβ plaques has long been investigated as a potential AD treatment. However, these Aβ-targeted interventions have had limited success in delaying cognitive decline even when Aβ removal was successful (Ackley et al., 2021).

Amyloid-β is a commonly synthesized and secreted protein (Seubert et al., 1992); however, in AD, two primary oligomers become dominant, Aβ40 and Aβ42. Aβ40 is produced in higher concentrations, though Aβ42 is most prevalent in plaques (Jarrett et al., 1993; Iwatsubo et al., 1994). In AD, Aβ42 aggregates into plaques, and the brain’s clearance system cannot clear the proteins into the CSF. This aggregation explains the decrease of Aβ42 in CSF in AD compared to Cognitively Normal (CN) controls while Aβ40 levels remain consistent across diagnoses (Lewczuk et al., 2004; Sturchio et al., 2021). Due to these established changes over time, the CSF Aβ40:Aβ42 ratio has been implicated as a good measure of AD progression. PET studies have demonstrated that utilizing this ratio is a more effective and reliable measure than using the decrease in CSF Aβ42 alone (Janelidze et al., 2016; Lewczuk et al., 2017).

**FIGURE 1:**
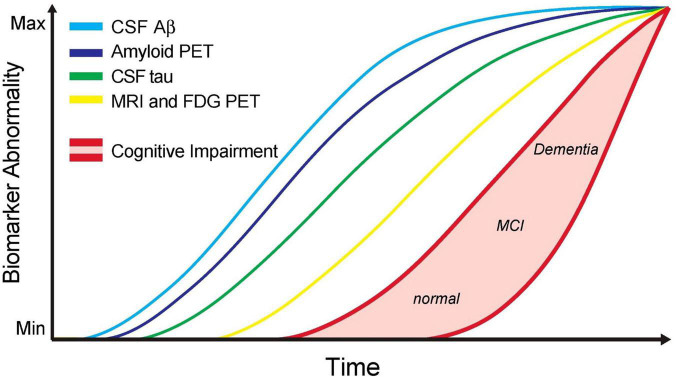
Updated model of the jack graph. In this new model, the ordering of some biomarkers has been altered to reflect a new understanding, and the horizontal axis now depicts time. As all curves converge in the top right of the graph, this indicates the point of maximum abnormality, and the red shaded area indicates a cognitive response to this abnormality. Cerebrospinal fluid (CSF) Aβ42 and amyloid-positron emission tomography (PET) measure amyloid-β burden. CSF tau to measure tau burden. MRI + FDG PET to measure neurodegeneration. Cognitive impairment with low and high-risk regions. Scale is biomarker abnormality, not concentration increase. Figure from Pawlowski et al. (2017).

It is well-established that Aβ accumulation begins many years prior to the onset of AD symptoms and that people can have an excess of Aβ in their brains without cognitive symptoms. Many other hypotheses in the literature attempt to explain the pathologic processes that occur through AD; however, the Aβ hypothesis has the most support currently (Liu et al., 2019). The Aβ hypothesis posits that the excess of Aβ initiates further pathogenic mechanisms that lead to the onset of neurodegeneration and disease symptoms (Hardy and Higgins, 1992). Despite the Aβ hypothesis being dominant for the last 30 years, excess Aβ in a substantial proportion of CN people and the failure of Aβ targeting therapies to impact AD suggest other mechanisms are at work. This theory has been elegantly illustrated by “the Jack curve” (Figure 1; Jack et al., 2013), whereby the onset of cognitive symptoms is temporally related to the increase in tau and brain atrophy long after the accumulation of Aβ.

### Tau pathology

In healthy brains, tau binds to microtubules to aid stability and promote axonal transport in neurons as a microtubule-associated protein (MAP) (Mandelkow and Mandelkow, 2012; Chang et al., 2021). This axonal stability is regulated through a balance between phosphorylation and dephosphorylation of tau (Wang et al., 1995). Disruption of this balance is observed in AD, and hyperphosphorylation of tau is able to occur (Luna-Muñoz et al., 2013). Phosphorylation of tau is known to affect its solubility, localization, function, interactions, and susceptibility to further post-translational modification (Luna-Muñoz et al., 2013). As such, it is suggested that abnormal phosphorylation causes the loss of tau’s positive charge, leading to a formation change and ultimately causing detachment from the microtubules (Mietelska-Porowska et al., 2014). Once detached, tau is free to move toward the neuronal soma, where it can accumulate as aggregated tau and eventually form NFTs (Mietelska-Porowska et al., 2014). There has also been a relationship observed between the post-translational truncation and phosphorylation of tau preceding aggregation in AD (Flores-Rodríguez et al., 2015). It appears that the tau pathogenesis in AD involves both hyperphosphorylation and predominantly N-terminal truncation of tau (Zhou et al., 2018).

The tau aggregation mechanism is still unknown; however, numerous findings suggest that aggregated tau acts as seeds that spread in a prion-like mechanism and trigger tau aggregation in surrounding cells (Wischik et al., 2018; Xu et al., 2022). This prion-like mechanism suggests that abnormal tau “seeds” from the donor transfer to a recipient, causing the recipient to become abnormal and go on to act like a donor (Wu et al., 2016). Accumulation of intraneuronal tau tangles is a common hallmark of AD (Wang and Mandelkow, 2016), and the seeding hypothesis may explain the regional spread that occurs in AD. This seeding hypothesis follows the Braak staging mechanism of NFTs (Braak and Braak, 1991).

**FIGURE 2:**
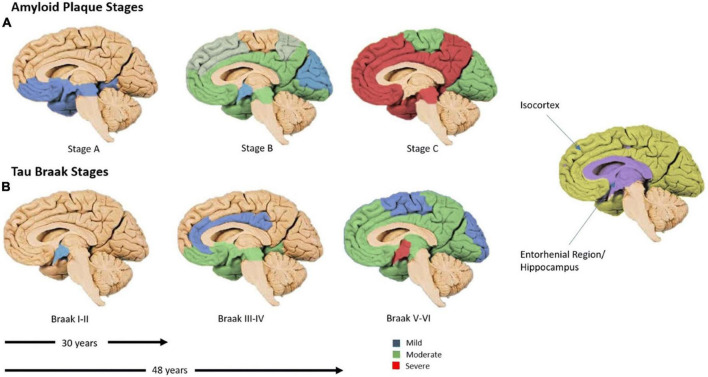
The time-course of amyloid-β and tau spread. (A) Amyloid-β spread, global by 30 years. (B) Regional spread of tau. The predominant appearance of tau in the temporal lobe before spreading outwards in cell-to-cell contacts (Spotorno et al., 2020). Six Braak stages (I–VI), tau burden, begins in the trans-entorhinal region (I–II), moves to the entorhinal region (III), then into the CA1 hippocampus (IV), before moving into almost all regions of the hippocampus and isocortex (V). Finally, these regions become severely affected (VI). Blue corresponds with mild tau burden, green with moderate and red with severe (Braak and Braak, 1991). Figure from Swarbrick et al. (2019).

Neurofibrillary tangles are the aggregates of hyperphosphorylated tau that form intra-neuronally before becoming extra-neuronal (Serrano-Pozo et al., 2011). Braak and Braak (1991) determined that tau tends to spread in a distinct pattern, beginning in the temporal lobe, progressing into the association cortices and finally into the sensorimotor cortices (Figure 2; Braak and Braak, 1991); subsequently, these postmortem findings were corroborated by tau-PET studies (Schöll et al., 2016).

Interestingly this set pattern of progression has also been closely associated with cognitive stage and disease progression long after Aβ has aggregated globally (Figure 2; Braak and Braak, 1991; Nelson et al., 2012; Schöll et al., 2016). As such, it is hypothesized that the toxic nature of tau arises from its ability to sequester normal MAPs when phosphorylated; this disrupts the microtubule network, eventually leading to neurodegeneration (Gong and Iqbal, 2008). When tau disruption becomes substantial, there is significant detachment from axons, allowing tau to be released into the CSF and NFT formation. Mounting evidence suggests that Aβ and tau work together in AD progression, rather than Aβ causing the aggregation of tau as hypothesized by the Aβ cascade hypothesis (Rapoport et al., 2002; Shipton et al., 2011; Busche and Hyman, 2020).

### The time-course of Alzheimer’s disease biomarkers

The relative time-course of these Aβ and tau pathologies and their contributions to AD presentation have been consolidated and presented by Jack et al. (2013) (Figure 1). Using the Alzheimer’s Disease Neuroimaging Initiative (ADNI) data to examine MCI patients who progressed to AD, this team showed that Aβ burden was not related to the risk of AD progression (Jack et al., 2010) and produced a widely referenced graph that depicts the time-course of different AD biomarkers, which has since been updated to include new findings (Jack et al., 2013).

To date, AD therapies have targeted each of the biomarkers of the Jack curve without success; therefore, alternative therapeutic targets (and biomarkers of disease progression) are needed. The glymphatic system presents a new diagnostic and therapeutic target for AD.

## Glymphatic system

The glymphatic system is the brain’s clearance pathway; this system removes waste products and transports solutes throughout the brain *via* CSF (Iliff et al., 2012). CSF makes up a large proportion of the total fluid volume within the mammalian head and flows throughout the four ventricles and subarachnoid space (Khasawneh et al., 2018). From the subarachnoid space, the CSF penetrates the brain parenchyma *via* the PVS (Khasawneh et al., 2018).

**FIGURE 3:**
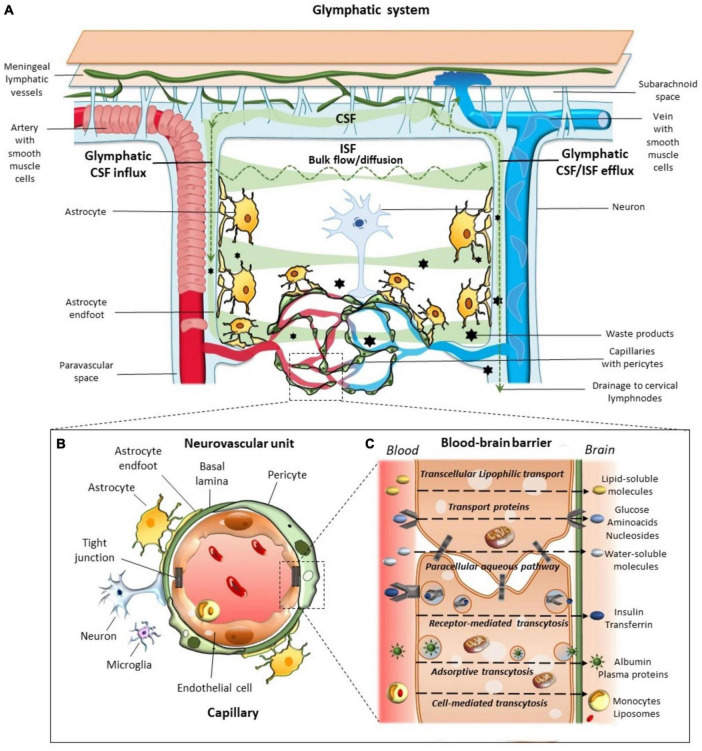
The glymphatic system. This system aids in the brain’s transport, movement, and removal of solutes. (A) Cerebrospinal fluid (CSF) enters *via* para-arterial spaces (red) and mixes with interstitial fluid (ISF) (Iliff et al., 2012). The CSF-ISF mix enters the para-venous space through the AQP4 channels and exits *via* the para-venous efflux pathway (blue) (Shetty and Zanirati, 2020). (B) Neurovascular Unit. Complex unit, with astrocyte end-feet enclosing the blood vessels (Sakai and Nishino, 2021). (C) Blood-Brain Barrier exchange of metabolites to maintain brain homeostasis (Keable et al., 2016). Figure from Natale et al. (2021).

Iliff et al. (2012) identified and labeled the glymphatic pathway, consolidating research that had observed lymphatic-like clearance in the brain (Figure 3A; Papaiconomou et al., 2004; Thrane et al., 2013). After flowing into the brain, CSF passes through AQP4 channels and mixes with interstitial fluid (ISF) (Figure 3; Shetty and Zanirati, 2020). AQP4 channels are selective water channels located on astrocyte end-feet that allow the mixing of CSF and ISF (Badaut et al., 2007). This mixture can then leave the brain through perivenous space and eventually out into the lymphatic vessels in the meninges and the lymphatics in the neck (Shetty and Zanirati, 2020).

Imaging studies have demonstrated the importance of this system in the clearance of Aβ and other proteins from the brain (Mestre et al., 2020). One such study demonstrated, through MRI and PET imaging, that ventricular CSF clearance was impaired in AD and related to increase Aβ deposits (de Leon et al., 2017). This team replicated this finding again in 2022 with a larger sample size (Li et al., 2022). Additionally, a recent study by Harrison et al. (2020) demonstrated that inhibition of Aquaporin-4 (AQP4) channels results in impaired glymphatic clearance, leading to impairment in tau clearance (Harrison et al., 2020).

Cerebrospinal fluid moves alongside the blood vessels in the brain; solutes, such as glucose, are delivered to regions where needed, while waste metabolites are removed to maintain homeostasis (Figure 3C; Iliff et al., 2013b). The CSF is propelled alongside the blood vessels through the pulsation of arterial walls (Iliff et al., 2013b; Mestre et al., 2018b). This pulsation was first observed by Iliff et al. (2012) through the use of two-photon microscopy in mouse models (Iliff et al., 2012, 2013b). The team labeled CSF with fluorescent tracers, which were injected into the cisterna magna of mice (Iliff et al., 2012). This study subsequently showed that CSF enters the brain along the cortical pial arteries, followed by CSF entry into the PVS along the penetrating arterioles (Figure 3B; Iliff et al., 2012). Crucially, this study showed that CSF did not spread diffusely or randomly in the parenchyma as previously believed; instead, it followed a pattern that mirrored the brain vasculature (Iliff et al., 2012).

Further studies are now beginning to demonstrate that impairment of the brain’s glymphatic drainage system is likely related to several neurodegenerative diseases (Iliff et al., 2012, 2014), including AD and related proteinopathies (Iliff et al., 2014). Iliff et al. (2012) also investigated whether Aβ was cleared along the perivascular pathway they had observed. Fluorescent Aβ was injected into the striatum of mice, which was then shown to be removed through para-venous efflux (Iliff et al., 2012). Furthermore, they found that AQP4 knockout mice showed a 65% reduction in CSF efflux compared to wild-type mice; this further reduced Aβ clearance by up to 55% (Iliff et al., 2012). These findings demonstrate the importance of an effective and functioning glymphatic system in Aβ clearance from the brain.

### Other Alzheimer’s disease biomarkers and the glymphatic system

Neurofilament light chain (NfL) is a subunit of neurofilaments that confer structural stability in neurons and allow the radial growth of axons (Hoffman et al., 1987; Eyer and Peterson, 1994). In AD and other neurodegenerative diseases, NfL is elevated in both the CSF and Plasma and therefore acts as a general marker of neurodegeneration (Lewczuk et al., 2018). Although these measures are a sensitive marker for neurodegeneration, they are not specific to AD (Beydoun et al., 2021). The relationship between NfL and glymphatic clearance in AD has yet to be investigated. Serum/CSF NfL ratio has been found to be lower in patients who have experienced subarachnoid hemorrhage versus controls which is hypothesized to be due to reduced glymphatic efflux (Garland et al., 2021). Another team showed that following subarachnoid hemorrhage, patients undergo AQP4 mislocalization, similar to those with AD, and show tau accumulation as the result of this mislocalization (Pu et al., 2019).

### Reduced glymphatic function risk factors

#### Apolipoprotein E4

**FIGURE 4:**
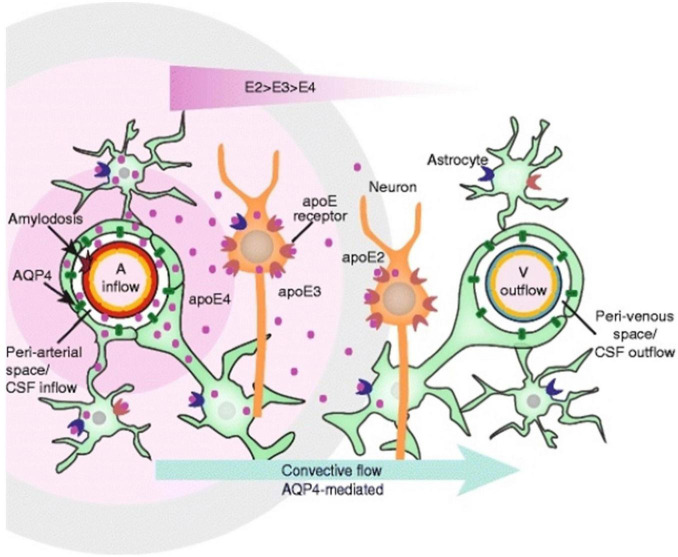
Apolipoprotein (APOE) distribution in the glymphatic system. This schematic depicts the convective flow that occurs around an artery and toward the venous space. APOE is responsible for lipid transport and repair in the brain (Liu et al., 2013). APOE is delivered around the arteries, and density depends on the alleles present, with APOE4 having the greatest density near the artery (A) (Achariyar et al., 2016). Figure from Achariyar et al. (2016).

Apolipoprotein E4 (APOE4) is the most decisive genetic risk factor for AD development (Corder et al., 1993), conferring both higher likelihood and earlier onset (William Rebeck et al., 1993). The APOE gene is responsible for lipid transport and repair in the brain as a cholesterol carrier (Liu et al., 2013). There are three dominant forms of the APOE gene, which vary in their ability to predispose an individual to AD. This gene exists as E2, E3, and E4 variants and has a frequency of 8.4, 77.9, and 13.7%, respectively (Figure 4; Farrer et al., 1997). The E4 allele predisposes one to an increased risk of AD development, four times higher for those who are heterozygous for the APOE4 mutation and twelve times higher for those homozygous for the APOE4 mutation (Holtzman et al., 2012). APOE4 carriers have increased cerebrovascular Aβ angiopathy due to increased deposition of Aβ at the vessel walls (Yu et al., 2015). This finding implicates APOE4 in the dysregulation of Aβ metabolism, allowing it to be deposited extracellularly as plaques, which supports previous findings (Schmechel et al., 1993; Polvikoski et al., 1995). Additionally, it has been shown that APOE4 carriers have increased degradation of the BBB and brain pericytes (Halliday et al., 2016).

#### Sleep

Sleep disturbance is a commonly reported symptom of AD patients (McCurry et al., 1999). Disturbance includes insomnia, poor sleep quality, or reduced sleep quantity (Irwin and Vitiello, 2019). Many papers have demonstrated the need for adequate sleep to allow glymphatic clearance (Ju et al., 2013; Kress et al., 2014; Ahmadian et al., 2018). It can therefore be hypothesized that sleep quality is critical for glymphatic clearance efficiency. A stand-out paper by Xie et al. (2013) used *in vivo* two-photon imaging to understand CSF influx in awake, anesthetized, and sleeping mice (Xie et al., 2013). The team discovered that the interstitial space in sleeping and anesthetized mice increased by 60%, resulting in a dramatic increase in convective exchange between CSF and ISF (Xie et al., 2013). This increased turnover resulted in an increased Aβ clearance during sleep, suggesting that sleep increases glymphatic system clearance (Xie et al., 2013). Ultimately, there is likely a bidirectional relationship between sleep and AD, in which poor sleep impacts the effectiveness of the glymphatic system, allowing Aβ and tau to accumulate, which worsens dementia symptoms and exacerbates sleep disturbances.

Furthermore, APOE has been implicated as an essential factor linking sleep disturbance and AD. A study by Drogos et al. (2016) determined that carriers of the APOE4 have decreased sleep quality, even without subjective sleep complaints (Drogos et al., 2016). Additionally, APOE4 carriers show a two-times increase in disordered breathing during sleep compared to non-carrier individuals (Kadotani et al., 2001). The increased incidence of AD among APOE4 carriers may result from their increased level of disordered sleep. Moreover, APOE4 carriers may have increased Aβ build-up in the brain’s sleep centers, and therefore it could be hypothesized that the body may begin to struggle to produce restorative sleep, compounding the issue. As such, a feedforward loop may be occurring with the driving factor of APOE4 leading to poor sleep, thus reducing glymphatic clearance, allowing Aβ build-up and driving worse sleep.

#### Aquaporin 4 water channel

**FIGURE 5:**
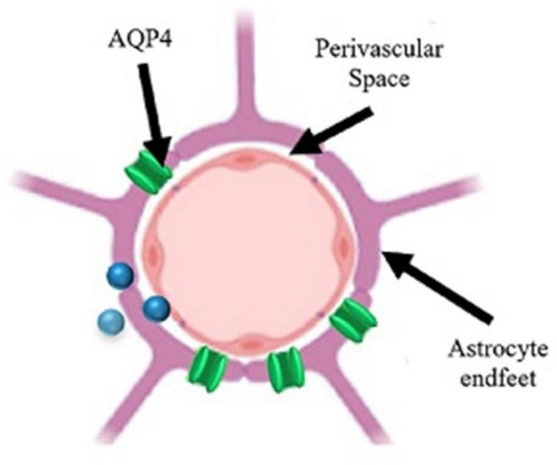
Schematic of a perivascular unit. Perivascular space (PVS) separates the astrocyte end-feet from the blood vessel. AQP4 (aquaporin-4 channel) allows for the influx/efflux of solutes into the PVS for removal or distribution. The inability of the cerebrospinal fluid (CSF) to flow through the PVS results in stagnation and enlargement of the PVS. Figure adapted from Troili et al. (2020).

Aquaporin-4 water channel channels are critical features of the glymphatic system, and their function is required to effectively remove toxins from the brain (Iliff et al., 2012). This selective water channel maintains ionic and osmotic homeostasis within the brain (Badaut et al., 2007). These channels are localized to astrocytes and ependymal cells, with the most extensive presence on the end-feet of perivascular astrocytes (Figure 5; Mathiisen et al., 2010). Loss of localization occurs when AQP4 loses its polarity and becomes broadly associated with the entire astrocyte (Murlidharan et al., 2016). This mislocalization of AQP4 is known to occur in normal aging and excessively in AD (Yang et al., 2011).

Aquaporin-4 water channel has been shown to play an important role in the removal of Aβ from the brain parenchyma (Rosu et al., 2020). One such study determining that inhibition of AQP4 decreases Aβ drainage from around cerebral vessels (Rosu et al., 2020). However, this paper did not investigate Aβ42, as Aβ40 is the soluble form of Aβ. However, one study was able to demonstrate, through postmortem histological analysis, that non-demented subjects showed a reduced end-feet AQP4 localization on capillaries that was associated with increases in soluble and insoluble Aβ40 and soluble Aβ42 (Simon et al., 2022). Furthermore, reduced end-feet AQP4 localization on large vessels was associated with increases in soluble Aβ40 and soluble Aβ42 (Simon et al., 2022). Although causality cannot be conferred as this is a postmortem study, these recent findings were only present in non-demented subjects (not those with AD) which suggests that AQP4 mislocalization is occurring early in the AD disease continuum.

Yang et al. (2011) inferred that Aβ accumulation is the cause, rather than the consequence, of AQP4 mislocalization in a mouse AD model (Yang et al., 2011). The team concluded that these Aβ deposits at the PVS were likely particularly harmful to cognition as they compromised end-foot function and, therefore, the homeostatic function of astrocytes (Yang et al., 2011). However, this finding remains controversial whether AQP4 mislocalization is the cause or consequence of Aβ deposition. Several other studies have shown that AQP4 mislocalization suppresses glymphatic clearance and is the cause of Aβ accumulation (Iliff et al., 2014; Kress et al., 2014; Zeppenfeld et al., 2017). Opposing findings such as this may suggest a feedforward relationship where Aβ can accumulate due to other problems with the glymphatic system, which causes AQP4 mislocalization, further promoting Aβ accumulation (Silva et al., 2021).

Additionally, AQP4 mutations have been shown to promote sleep disturbances (Rainey-Smith et al., 2018). A study by Rainey-Smith et al. (2018) utilized a self-reported “overall” sleep quality assessment and demonstrated that several AQP4 single nucleotide polymorphisms were associated with poorer sleep (Rainey-Smith et al., 2018). Another recent study on Parkinson’s disease demonstrated that specific mutations in AQP4 were likely the cause of sleep disturbance and may act as a prognostic marker for cognitive decline (Fang et al., 2022). Conversely, some studies have found that sleep disturbance results in AQP4 mislocalization; one such study found that mice who were sleep deprived showed AQP4 mislocalization away from astrocyte end-feet (Liu et al., 2017). In agreement with this finding, Zhang et al. (2020) found that short-term sleep deprivation (1-week) causes abnormal expression of AQP4 and a reduction in glymphatic clearance (Zhang et al., 2020). So, again, it is unclear if AQP4 mislocalization causes poor sleep or if poor sleep causes abnormal expression of AQP4.

The APOE4 and AQP4 haplotypes may lie very early on the Jack curve (Figure 1). Genetic variants in AQP4 and APOE4, which influence gene expression and increase the risk of progression to dementia, may help explain individual differences in susceptibility to cognitive decline and dementia in the context of sleep (Rainey-Smith et al., 2018). These alterations in normal biology may ultimately disrupt the effectiveness of the glymphatic system, promoting an individual’s vulnerability to developing AD. This relationship warrants further investigation to gain clarification and understand whether these markers could aid in early intervention and treatment.

#### Aging

**FIGURE 6:**
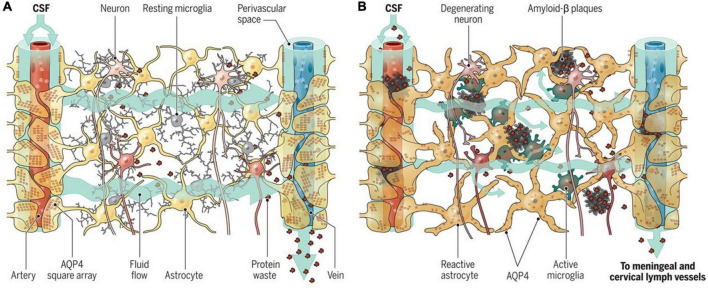
Aging and the glymphatic system. (A) Adequate influx and efflux of cerebrospinal fluid (CSF) and interstitial fluid (ISF) is needed to remove unwanted metabolites from the brain. (B) With aging, glymphatic dysfunction begins to occur, which contributes to the build-up of molecules known to be associated with neurodegeneration (Da Mesquita et al., 2018). Figure reproduced with permission from Nedergaard and Goldman (2020).

Finally, aging is known to significantly influence the development of neurodegenerative diseases, such as AD (Figure 6). With aging comes degeneration in neurons, Aβ plaques, astrocyte and microglia dysfunction and AQP4 mislocalization, ultimately leading to a reduction in protein waste removal (Figure 6; Nedergaard and Goldman, 2020). Moreover, aging is related to a drastic decline in the efficiency of exchange between CSF and the brain parenchyma in mice (Kress et al., 2014). Older mice had a 40% reduction in Aβ clearance compared to young mice and a 27% loss in vessel pulse ability and loss of AQP4 polarization (Kress et al., 2014). Thus, the team proposed that cognitive decline associated with aging is also associated with impaired glymphatic clearance and may be a target for neurodegenerative disorder treatment (Kress et al., 2014). Furthermore, findings such as this may suggest that the elasticity of PVS is significantly decreased with age, and a reduction in pulse ability of the PVS may suggest why age is associated with enlarged PVS.

## Perivascular spaces as a biomarker for Alzheimer’s disease

Part of the glymphatic pathway, PVS are the small fluid-filled spaces through which CSF flows in and out of the brain. These spaces surround arteries, arterioles, capillaries, veins, and venule walls as they pass from the subarachnoid space to the parenchyma (Jessen et al., 2015).

Iliff et al. (2013a) discovered that PVS are molecular size/weight dependent. Two molecules of differing molecular weight were injected into the rat intrathecal space and observed with MRI. Both molecules passed through the para-arterial space, but the smaller molecule entered more extensively into the brain parenchyma (Iliff et al., 2013a). Interestingly, this finding points toward a size-selective nature of the PVS when clearing molecules from the brain. Moreover, this may suggest why large molecules found in AD, such as Aβ plaques, are deposited around blood vessels, as they simply cannot enter and be clear from the PVS (Yu et al., 2022).

**FIGURE 7:**
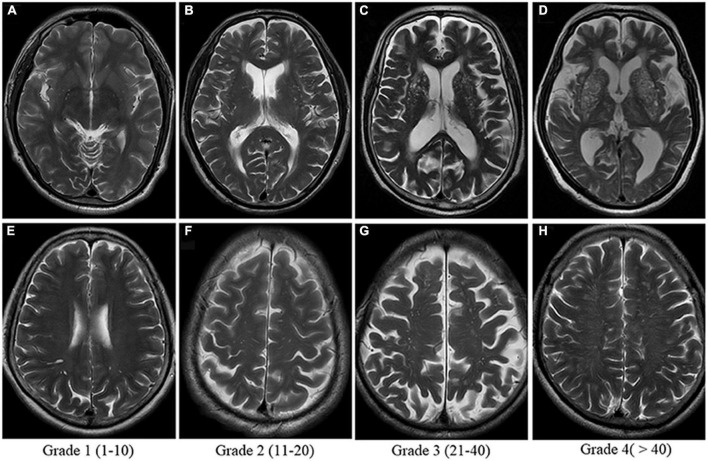
Enlarged perivascular space (PVS) visual score rating as shown on T2-weighted MRI. Grades 2, 3, and 4 are considered abnormally enlarged. Panels (A–D) show severity of enlarged PVS in the Basal Ganglia. Panels (E–H) show severity of enlarged PVS in the centrum semi-ovale. The Basal Ganglia and Centrum Semi-Ovale are currently implicated as the most accurate measures of PVS burden. Image from Yu et al. (2022).

Stagnation of the PVS occurs when there is insufficient flow of CSF through these spaces, causing them to expand, trap fluid and become visible on MRI (Figure 7; Wardlaw et al., 2020). Although enlarged PVS (ePVS) are a typical sign of aging, it is also established that neurodegeneration increases the prevalence and severity of ePVS (Chen et al., 2011). ePVS may function as indicators for disease initiation and progression as these spaces must be functional to maintain homeostasis and remove excess metabolites, such as those that form Aβ plaques and NFTs (Keable et al., 2016). Peng et al. (2016) showed that glymphatic clearance issues preceded Aβ deposition and, therefore, may precede Aβ in the AD disease continuum (Peng et al., 2016). The team utilized a radiolabeled approach and intracisternal injection of Aβ40 and inulin (reference molecule) to determine that Aβ influx was reduced with aging and in mice models of AD (Peng et al., 2016). The same approach then quantified Aβ clearance, and it was found that there was greater inulin clearance compared to Aβ; this suggests that the slower clearance time of Aβ increases the time that Aβ can interact with endogenous Aβ plaques, promoting further accumulation (Peng et al., 2016). While this is not a direct measurement of glymphatic problems preceding Aβ accumulation, the team believed that the data suggested that glymphatic clearance is altered in AD and occurs before the significant presence of Aβ (Peng et al., 2016). This team also hypothesized that restoring glymphatic efficiency and PVS dynamics may be a potential treatment for slowing AD progression (Peng et al., 2016).

Banerjee et al. (2017) supported this finding and hypothesized that centrum semi-ovale PVS clearance was essential for Aβ clearance within the brain through their analysis of Aβ-PET and MRI (Banerjee et al., 2017). ePVS were quantified using a pre-defined slice from a T1/2/2* MRI and FLAIR (Fluid attenuated inversion recovery) images (Banerjee et al., 2017). An adjusted analysis found that the severity of ePVS in the centrum semi-ovale was associated with AD, while the severity of ePVS in the basal ganglia was associated with subcortical vascular cognitive impairment (Banerjee et al., 2017). However, they did not find an association between MRI visible ePVS and Aβ-PET (using Pittsburgh compound B). This team noted that their study would have been improved with the use of CSF biomarkers of Aβ to ensure that the Aβ-PET signal was not measuring only parenchymal Aβ but also vascular Aβ (Banerjee et al., 2017). Nonetheless, this study gives supporting evidence for using ePVS in the centrum semi-ovale as an imaging marker for AD, although it is not likely a measure of Aβ-PET positivity in patients (Banerjee et al., 2017).

Barisano et al. (2021) also demonstrated in an extensive review of subjects using the Human Connectome Project that the PVS are smaller in the morning and bigger in the afternoon in the same subjects (Barisano et al., 2021). This finding suggests a diurnal variation in the caliber of the PVS, which are smaller during sleep and enlarged during the day. Furthermore, this ability to change in caliber decreases as neurovascular compliance decreases with aging, so the PVS will gradually lose the ability to change in caliber as we age and remain permanently dilated (Barisano et al., 2021).

### Amyloid-beta and perivascular spaces

It has been demonstrated that the glymphatic pathway and associated PVS are important in Aβ clearance. Studies by Xu et al. (2015) utilized AQP4 knockout mice to demonstrate the importance of Aβ clearance in AD (Xu et al., 2015). These knockout mice could not effectively clear Aβ, resulting in memory deficits (Xu et al., 2015). These results were supported by Mestre et al. (2018a), who found that AQP4 knockout mice had reduced CSF influx into the PVS (Mestre et al., 2018a). Furthermore, the disruption of AQP4 localization, as seen in AD, can increase Aβ pathology (Simon et al., 2022). Issues with AQP4 are associated with AD pathology and cognitive function, suggesting that AQP4 localization may be a key driver in disease progression (Wilcock et al., 2009; Zeppenfeld et al., 2017; Simon et al., 2022).

Another recent study determined, using *ex vivo* MRI on humans, that ePVS were located mainly in the white matter portion of perforating cortical arterioles (Perosa et al., 2021). They showed reduced small muscle cells and increased vascular Aβ that extended into the white matter, which individually affected vessels with ePVS (Perosa et al., 2021). Thus, their results were consistent with the current theory that ePVS reflect an impaired outward flow along the arterioles that PVS follow (Perosa et al., 2021).

### Tau and perivascular spaces

Studies have demonstrated that both p-tau and total-tau (t-tau) burden are associated with ePVS. Many believe that tau is cleared through intracellular degradation (Tarasoff-Conway et al., 2015); however, upon being released into the interstitium (by AD-related neuronal death), it can then be cleared by the glymphatic system (Iliff et al., 2014).

Vilor-Tejedor et al. (2021) used 322 CN individuals diagnosed with AD-like pathology demonstrating that ePVS in the centrum semi-ovale was associated with elevated CSF p-tau and t-tau (Vilor-Tejedor et al., 2021). Another study determined that NFT pathology was a good correlate for ePVS burden in patients with AD and vascular lesion AD compared to controls (Boespflug et al., 2018). By measuring postmortem ePVS and AQP4 expression using immunofluorescence, this team also found that AD patients had increased AQP4 expression but a reduced localization to the astrocyte end-feet (Boespflug et al., 2018). These patients also showed an increased tau and Aβ burden associated with an increase in ePVS (Boespflug et al., 2018). Both studies also support Iliff et al. (2014), who determined that following traumatic brain injury, mice lacking the AQP4 channel showed an increase in NFT pathology and neurodegeneration (Iliff et al., 2014). A more recent study in normal patients with AD pathological biomarkers utilized an ePVS visual rating score to suggest that impairment in glymphatic clearance could contribute to tau accumulation through microglia neuroinflammation processes (Zeng et al., 2022).

In a large cross-section study, PVS distribution was found to be different in individuals with MCI compared to CN participants, with a higher PVS volume fraction in the centrum semi-ovale of the white matter (Sepehrband et al., 2021). Further, it was shown that a lower PVS volume fraction in the medial temporal lobe was correlated to a more significant aggregation of tau NFTs in the adjacent entorhinal cortex (Sepehrband et al., 2021). The team hypothesized that the observed decrease in PVS volume fraction in the medial temporal lobe might represent occlusion of PVS prior to enlargement (Sepehrband et al., 2021).

In opposition to these findings, Gertje et al. (2021) found no significant association between ePVS and AD (Gertje et al., 2021). In 39 individuals with AD, CSF levels of Aβ42, p-tau, t-tau, neuroinflammatory markers, and Aβ-PET were not associated with PVS (Gertje et al., 2021).

To date, the role of ePVS in the development and progression of AD has yet to be established. We hypothesize that the impairment of glymphatic clearance *via* perivascular spaces is an early pathophysiologic mechanism that leads to Aβ and tau deposition in the brain. The interaction between ePVS, AD, and associated genetic and lifestyle risk factors needs further elucidation to determine the potential of ePVS as both an early diagnostic biomarker of neurodegeneration and a target for more effective therapeutic interventions.

## Conclusion

In conclusion, the glymphatic system and its associated PVS are of great interest in AD. Understanding more about AD and its underlying mechanisms is key to early intervention and effective treatment. Recent findings regarding APOE4 and AQP4 haplotypes, as well as the effect of sleep on PVS, demonstrate the complex nature of this neurodegenerative disease. Enlarged PVS have the potential as a biomarker able to predict the chance of neurodegeneration through to AD; additionally, they may be able to demonstrate disease progression and severity. Furthermore, these spaces and the glymphatic system more generally may be a potential target for treatment, with the promotion of glymphatic clearance potentially delaying AD-associated cognitive decline.

## Author contributions

MLy wrote the manuscript. MLy, MLa, and LV conceived the scope of the review. All authors contributed to edits, revisions, and approval for submission.

## Abbreviations

AD: Alzheimer’s disease
Aβ: amyloid-beta
PVS: perivascular space
MCI: mild cognitive impairment
NFT: neurofibrillary tangle
p-tau: phosphorylated-tau
APP: amyloid precursor protein
PS: presenilin
CSF: cerebrospinal fluid
CN: cognitively normal
MAP: microtubule-associated protein
PET: positron emission tomography
ADNI: Alzheimer’s disease neuroimaging initiative
AQP4: aquaporin-4 water channel
ISF: interstitial fluid
NfL: neurofilament light-chain
APOE4: apolipoprotein E gene
BBB: blood-brain barrier
MRI: magnetic resonance imaging
ePVS: enlarged perivascular space
t-tau: total-tau.
